# 3,3-Dichloro-1-ethyl-1*H*-2,1-benzothia­zin-4(3*H*)-one 2,2-dioxide

**DOI:** 10.1107/S1600536809003079

**Published:** 2009-01-31

**Authors:** Muhammad Shafiq, M. Nawaz Tahir, Islam Ullah Khan, Saeed Ahmad, Muhammad Nadeem Arshad

**Affiliations:** a Government College University, Department of Chemistry, Lahore, Pakistan; b University of Sargodha, Department of Physics, Sargodha, Pakistan; c Department of Chemistry, University of Science and Technology Bannu, Pakistan

## Abstract

In the title compound, C_10_H_9_Cl_2_NO_3_S, the S atom, which is a component atom of a heterocyclic ring, shows tetra­hedral coordination. The heterocyclic ring is not planar.

## Related literature

For related compounds, see: Arshad *et al.* (2008 ▶); Shafiq, Khan *et al.* (2008 ▶); Shafiq, Tahir *et al.* (2008 ▶); Tahir *et al.* (2008 ▶).
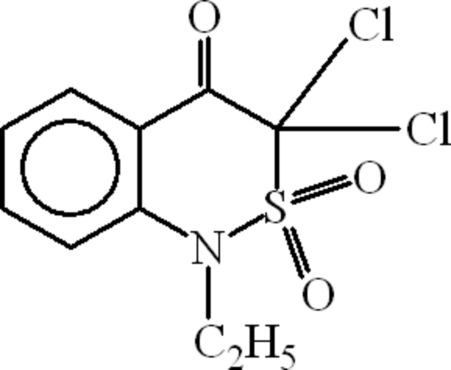


## Experimental

### 

#### Crystal data

C_10_H_9_Cl_2_NO_3_S
*M* *_r_* = 294.14
Monoclinic, 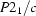

*a* = 7.7416 (2) Å
*b* = 11.9185 (3) Å
*c* = 12.9614 (3) Å
β = 95.995 (2)°
*V* = 1189.39 (5) Å^3^
*Z* = 4
Mo *K*α radiation
μ = 0.72 mm^−1^
*T* = 296 (2) K
0.24 × 0.20 × 0.18 mm

#### Data collection

Bruker Kappa APEXII CCD diffractometer
Absorption correction: multi-scan (*SADABS*; Bruker, 2005 ▶) *T*_min_ = 0.838, *T*_max_ = 0.881
12499 measured reflections
3082 independent reflections
1872 reflections with *I* > 2σ(*I*)
*R*_int_ = 0.041

#### Refinement

*R*[*F* ^2^ > 2σ(*F* ^2^)] = 0.042
*wR*(*F* ^2^) = 0.110
*S* = 1.01
3082 reflections
154 parameters
H-atom parameters constrained
Δρ_max_ = 0.41 e Å^−3^
Δρ_min_ = −0.28 e Å^−3^

### 

Data collection: *APEX2* (Bruker, 2007 ▶); cell refinement: *APEX2*; data reduction: *SAINT* (Bruker, 2007 ▶); program(s) used to solve structure: *SHELXS97* (Sheldrick, 2008 ▶); program(s) used to refine structure: *SHELXL97* (Sheldrick, 2008 ▶); molecular graphics: *ORTEP-3 for Windows* (Farrugia, 1997 ▶) and *PLATON* (Spek, 2003 ▶); software used to prepare material for publication: *WinGX* (Farrugia, 1999 ▶) and *PLATON*.

## Supplementary Material

Crystal structure: contains datablocks global, I. DOI: 10.1107/S1600536809003079/ng2539sup1.cif

Structure factors: contains datablocks I. DOI: 10.1107/S1600536809003079/ng2539Isup2.hkl

Additional supplementary materials:  crystallographic information; 3D view; checkCIF report

## Figures and Tables

**Fig. 1.**
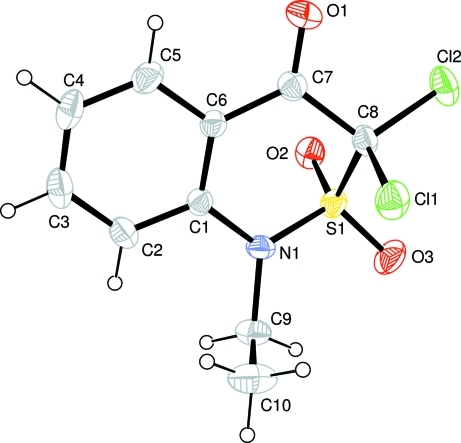
ORTEP drawing of the title compound, with the atom numbering scheme. The thermal ellipsoids are drawn at the 30% probability level. H-atoms are shown by small circles of arbitrary radii. The dotted lines show the intramolecular H-bonds.

**Fig. 2.**
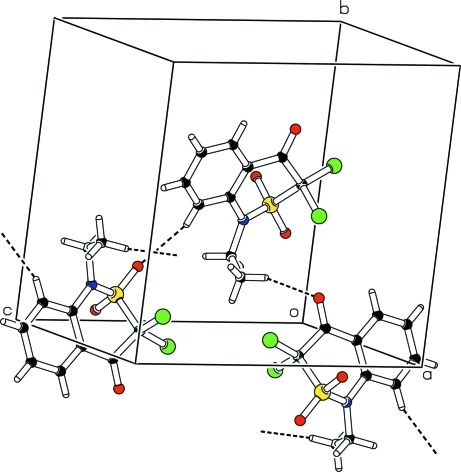
The partial packing figure (PLATON: Spek, 2003) which shows that molecules are connected through intermolecular H-bonds along the c axis in helical way.

## supplementary crystallographic information

### Comment

In continuation to the formation of different 2,1-Benzothiazine (Shafiq, Khan *et al.*, 2008), (Tahir *et al.*, 2008), (Arshad *et al.*, 2008), the title compound (I), (Fig 1), has been prepared.

We compare the bond distances and bond angles realised in (I) with the corresponding values observed in 3,3-Dibromo-1-ethyl-1*H*-2,1-benzothiazin- 4(3*H*)-one 2,2-dioxide (II) (Shafiq, Tahir *et al.*, 2008), which is structural isomer of (I). The bond distances S1—C8 [1.817 (2) Å] and S1—N1 [1.625 (2) Å] are larger as compared to 1.792 (8) and 1.617 (6) Å, respectively. This change in the thiazine ring is observed due to the reduction of C–Cl [1.744 (2), 1.766 (2) Å] bonds as compared with C—Br [1.898 (7), 1.947 (8) Å] bonds. The dihedral angle of benzene ring with *N*-ethyl moiety and the SO_2_ group is 78.08 (25)° and 77.99 (11)°, respectively. There exist intermolecular H-bonds (Table 1), due to which the molecules are connected in helical way along the *c* axis.

### Experimental

The title copound was prepared following the same method as in Shafiq, Tahir *et al.* (2008). A mixture of 1-Ethyl-1*H*-2,1 benzothiazin-4(3*H*)-one 2,2 dioxide (Shafiq, Khan *et al.*, 2008)(34 mg, 0.151 mmol), *N*-Chloro Succinamide (40.2 mg, 0.302 mmol) and Benzoylperoxide (2.11 mg, 0.009 mmol) in Carbon Tetra Chloride (10 ml), was heated under reflux for two hours. CCl_4_ was evaporated under reduced pressure and the residue was recrystallized in ethanol for X-ray diffraction studies.

### Crystal data

**Table:**

C_10_H_9_Cl_2_NO_3_S	*F*(000) = 600
*M**_r_* = 294.14	*D*_x_ = 1.643 Mg m^−^^3^
Monoclinic, *P*2_1_/*c*	Mo *K*α radiation, λ = 0.71073 Å
Hall symbol: -P 2ybc	Cell parameters from 3082 reflections
*a* = 7.7416 (2) Å	θ = 2.3–28.7°
*b* = 11.9185 (3) Å	µ = 0.72 mm^−^^1^
*c* = 12.9614 (3) Å	*T* = 296 K
β = 95.995 (2)°	Prismatic, colorless
*V* = 1189.39 (5) Å^3^	0.24 × 0.20 × 0.18 mm
*Z* = 4	

### Data collection

**Table:**

Bruker Kappa APEXII CCD diffractometer	3082 independent reflections
Radiation source: fine-focus sealed tube	1872 reflections with *I* > 2σ(*I*)
graphite	*R*_int_ = 0.041
Detector resolution: 7.40 pixels mm^-1^	θ_max_ = 28.7°, θ_min_ = 2.3°
ω scans	*h* = −10→10
Absorption correction: multi-scan (*SADABS*; Bruker, 2005)	*k* = −16→16
*T*_min_ = 0.838, *T*_max_ = 0.881	*l* = −17→15
12499 measured reflections	

### Refinement

**Table:**

Refinement on *F*^2^	Primary atom site location: structure-invariant direct methods
Least-squares matrix: full	Secondary atom site location: difference Fourier map
*R*[*F*^2^ > 2σ(*F*^2^)] = 0.042	Hydrogen site location: inferred from neighbouring sites
*wR*(*F*^2^) = 0.110	H-atom parameters constrained
*S* = 1.01	*w* = 1/[σ^2^(*F*_o_^2^) + (0.046*P*)^2^ + 0.3265*P*] where *P* = (*F*_o_^2^ + 2*F*_c_^2^)/3
3082 reflections	(Δ/σ)_max_ < 0.001
154 parameters	Δρ_max_ = 0.41 e Å^−^^3^
0 restraints	Δρ_min_ = −0.28 e Å^−^^3^

### Special details

**Table:**

Geometry. Bond distances, angles *etc*. have been calculated using the rounded fractional coordinates. All su's are estimated from the variances of the (full) variance-covariance matrix. The cell e.s.d.'s are taken into account in the estimation of distances, angles and torsion angles
Refinement. Refinement of *F*^2^ against ALL reflections. The weighted *R*-factor *wR* and goodness of fit *S* are based on *F*^2^, conventional *R*-factors *R* are based on *F*, with *F* set to zero for negative *F*^2^. The threshold expression of *F*^2^ > σ(*F*^2^) is used only for calculating *R*-factors(gt) *etc*. and is not relevant to the choice of reflections for refinement. *R*-factors based on *F*^2^ are statistically about twice as large as those based on *F*, and *R*- factors based on ALL data will be even larger.

### Fractional atomic coordinates and isotropic or equivalent isotropic displacement parameters (Å^2^)

**Table:**

	*x*	*y*	*z*	*U*_iso_*/*U*_eq_	
Cl1	0.45866 (9)	0.41954 (6)	0.18953 (6)	0.0592 (3)	
Cl2	0.21735 (10)	0.55261 (7)	0.05418 (5)	0.0649 (3)	
S1	0.08395 (8)	0.40174 (5)	0.20115 (5)	0.0421 (2)	
O1	0.2953 (3)	0.68290 (16)	0.23915 (16)	0.0637 (8)	
O2	−0.0624 (2)	0.47361 (15)	0.19971 (13)	0.0498 (6)	
O3	0.0781 (3)	0.30825 (16)	0.13333 (15)	0.0635 (7)	
N1	0.1508 (3)	0.36020 (16)	0.31795 (15)	0.0420 (7)	
C1	0.1973 (3)	0.4423 (2)	0.39506 (17)	0.0359 (7)	
C2	0.1918 (3)	0.4159 (2)	0.49864 (19)	0.0472 (9)	
C3	0.2348 (4)	0.4945 (3)	0.5745 (2)	0.0579 (10)	
C4	0.2803 (4)	0.6011 (3)	0.5502 (2)	0.0592 (10)	
C5	0.2877 (3)	0.6290 (2)	0.4482 (2)	0.0510 (9)	
C6	0.2506 (3)	0.5507 (2)	0.36908 (18)	0.0369 (7)	
C7	0.2704 (3)	0.5872 (2)	0.26343 (19)	0.0418 (8)	
C8	0.2613 (3)	0.4951 (2)	0.17788 (17)	0.0419 (8)	
C9	0.1481 (4)	0.2393 (2)	0.3441 (2)	0.0549 (10)	
C10	0.3251 (4)	0.1952 (3)	0.3774 (3)	0.0811 (14)	
H2	0.15879	0.34423	0.51707	0.0567*	
H3	0.23274	0.47473	0.64379	0.0693*	
H4	0.30605	0.65411	0.60215	0.0711*	
H5	0.31804	0.70172	0.43143	0.0612*	
H9A	0.09821	0.19756	0.28397	0.0658*	
H9B	0.07472	0.22786	0.39944	0.0658*	
H10A	0.31778	0.11692	0.39385	0.1217*	
H10B	0.37431	0.23552	0.43749	0.1217*	
H10C	0.39743	0.20468	0.32215	0.1217*	

### Atomic displacement parameters (Å^2^)

**Table:**

	*U*^11^	*U*^22^	*U*^33^	*U*^12^	*U*^13^	*U*^23^
Cl1	0.0513 (4)	0.0709 (5)	0.0564 (4)	0.0130 (3)	0.0109 (3)	0.0003 (4)
Cl2	0.0770 (5)	0.0835 (6)	0.0339 (3)	0.0038 (4)	0.0045 (3)	0.0121 (3)
S1	0.0482 (3)	0.0423 (4)	0.0347 (3)	−0.0006 (3)	−0.0011 (3)	−0.0072 (3)
O1	0.0897 (15)	0.0416 (12)	0.0612 (13)	−0.0127 (10)	0.0142 (11)	0.0090 (10)
O2	0.0442 (10)	0.0585 (12)	0.0450 (10)	0.0060 (8)	−0.0028 (8)	−0.0015 (9)
O3	0.0797 (14)	0.0552 (12)	0.0542 (12)	−0.0042 (10)	−0.0001 (10)	−0.0249 (10)
N1	0.0558 (13)	0.0286 (11)	0.0403 (12)	−0.0035 (9)	−0.0005 (9)	0.0020 (9)
C1	0.0369 (12)	0.0374 (13)	0.0327 (12)	0.0025 (10)	0.0006 (9)	−0.0007 (10)
C2	0.0503 (14)	0.0531 (17)	0.0383 (14)	0.0035 (12)	0.0047 (11)	0.0094 (12)
C3	0.0592 (18)	0.082 (2)	0.0317 (14)	0.0094 (16)	0.0013 (12)	−0.0010 (14)
C4	0.0635 (18)	0.073 (2)	0.0393 (16)	0.0020 (16)	−0.0035 (13)	−0.0213 (15)
C5	0.0576 (17)	0.0452 (16)	0.0488 (16)	−0.0049 (12)	−0.0008 (12)	−0.0114 (13)
C6	0.0403 (13)	0.0362 (13)	0.0336 (12)	0.0004 (10)	0.0004 (10)	−0.0020 (10)
C7	0.0415 (13)	0.0429 (15)	0.0408 (14)	−0.0032 (11)	0.0032 (10)	0.0014 (12)
C8	0.0464 (14)	0.0494 (15)	0.0299 (13)	0.0041 (11)	0.0034 (10)	0.0034 (11)
C9	0.0631 (18)	0.0348 (15)	0.0662 (19)	−0.0044 (13)	0.0045 (14)	0.0063 (13)
C10	0.075 (2)	0.0483 (19)	0.121 (3)	0.0103 (16)	0.015 (2)	0.0158 (19)

### Geometric parameters (Å, °)

**Table:**

Cl1—C8	1.766 (2)	C5—C6	1.394 (3)
Cl2—C8	1.744 (2)	C6—C7	1.460 (3)
S1—O2	1.4189 (18)	C7—C8	1.557 (3)
S1—O3	1.417 (2)	C9—C10	1.489 (4)
S1—N1	1.625 (2)	C2—H2	0.9300
S1—C8	1.817 (2)	C3—H3	0.9300
O1—C7	1.204 (3)	C4—H4	0.9300
N1—C1	1.418 (3)	C5—H5	0.9300
N1—C9	1.481 (3)	C9—H9A	0.9700
C1—C2	1.384 (3)	C9—H9B	0.9700
C1—C6	1.408 (3)	C10—H10A	0.9600
C2—C3	1.374 (4)	C10—H10B	0.9600
C3—C4	1.364 (5)	C10—H10C	0.9600
C4—C5	1.370 (4)		
			
Cl1···O1	3.469 (2)	C2···C3^iv^	3.506 (4)
Cl1···O3	3.244 (2)	C2···C2^iv^	3.586 (3)
Cl1···N1	3.127 (2)	C3···C2^iv^	3.506 (4)
Cl1···C1	3.520 (2)	C3···O2^iv^	3.363 (3)
Cl2···O3	3.306 (2)	C3···C1^iv^	3.491 (4)
Cl2···O1	2.867 (2)	C6···O2	3.229 (3)
Cl2···O2	3.1604 (18)	C9···O2^viii^	3.273 (3)
Cl2···O2^i^	3.3972 (18)	C10···C2	3.285 (4)
Cl1···H10C	3.1500	C10···O2^viii^	3.418 (4)
Cl2···H10A^ii^	3.0600	C1···H10B	2.8500
O1···Cl1	3.469 (2)	C2···H10B	2.7400
O1···Cl2	2.867 (2)	C2···H9B	2.6900
O2···Cl2	3.1604 (18)	C9···H2	2.5600
O2···C6	3.229 (3)	C10···H2	2.9300
O2···Cl2^i^	3.3972 (18)	H2···C9	2.5600
O2···C10^iii^	3.418 (4)	H2···C10	2.9300
O2···C3^iv^	3.363 (3)	H2···H9B	2.1100
O2···C9^iii^	3.273 (3)	H2···H10B	2.4300
O3···C2^ii^	3.359 (3)	H2···O3^vii^	2.4800
O3···Cl2	3.306 (2)	H3···O2^iv^	2.6100
O3···Cl1	3.244 (2)	H4···O1^ix^	2.6400
O1···H5	2.4900	H5···O1	2.4900
O1···H10C^v^	2.6000	H9A···O3	2.3500
O1···H4^vi^	2.6400	H9A···O2^viii^	2.6900
O2···H3^iv^	2.6100	H9B···C2	2.6900
O2···H9A^iii^	2.6900	H9B···H2	2.1100
O2···H10A^iii^	2.7900	H10A···O2^viii^	2.7900
O3···H9A	2.3500	H10A···Cl2^vii^	3.0600
O3···H2^ii^	2.4800	H10B···C1	2.8500
N1···Cl1	3.127 (2)	H10B···C2	2.7400
C1···Cl1	3.520 (2)	H10B···H2	2.4300
C1···C3^iv^	3.491 (4)	H10C···Cl1	3.1500
C2···O3^vii^	3.359 (3)	H10C···O1^x^	2.6000
C2···C10	3.285 (4)		
			
O2—S1—O3	119.52 (12)	Cl1—C8—C7	108.92 (16)
O2—S1—N1	111.86 (11)	Cl2—C8—S1	108.36 (12)
O2—S1—C8	104.14 (11)	Cl2—C8—C7	111.57 (17)
O3—S1—N1	108.95 (11)	S1—C8—C7	107.00 (15)
O3—S1—C8	110.75 (12)	N1—C9—C10	112.0 (2)
N1—S1—C8	99.71 (11)	C1—C2—H2	120.00
S1—N1—C1	118.62 (16)	C3—C2—H2	120.00
S1—N1—C9	119.93 (16)	C2—C3—H3	119.00
C1—N1—C9	121.25 (19)	C4—C3—H3	119.00
N1—C1—C2	119.7 (2)	C3—C4—H4	120.00
N1—C1—C6	121.6 (2)	C5—C4—H4	120.00
C2—C1—C6	118.7 (2)	C4—C5—H5	119.00
C1—C2—C3	120.6 (2)	C6—C5—H5	119.00
C2—C3—C4	121.3 (2)	N1—C9—H9A	109.00
C3—C4—C5	119.1 (3)	N1—C9—H9B	109.00
C4—C5—C6	121.4 (2)	C10—C9—H9A	109.00
C1—C6—C5	118.8 (2)	C10—C9—H9B	109.00
C1—C6—C7	124.1 (2)	H9A—C9—H9B	108.00
C5—C6—C7	117.2 (2)	C9—C10—H10A	109.00
O1—C7—C6	124.2 (2)	C9—C10—H10B	110.00
O1—C7—C8	118.6 (2)	C9—C10—H10C	109.00
C6—C7—C8	117.2 (2)	H10A—C10—H10B	110.00
Cl1—C8—Cl2	111.28 (13)	H10A—C10—H10C	109.00
Cl1—C8—S1	109.60 (13)	H10B—C10—H10C	109.00
			
O2—S1—N1—C1	−57.1 (2)	N1—C1—C2—C3	−179.4 (2)
O2—S1—N1—C9	117.7 (2)	C6—C1—C2—C3	1.0 (4)
O3—S1—N1—C1	168.51 (19)	N1—C1—C6—C5	177.4 (2)
O3—S1—N1—C9	−16.6 (2)	N1—C1—C6—C7	−2.8 (4)
C8—S1—N1—C1	52.5 (2)	C2—C1—C6—C5	−3.1 (3)
C8—S1—N1—C9	−132.7 (2)	C2—C1—C6—C7	176.8 (2)
O2—S1—C8—Cl1	174.51 (11)	C1—C2—C3—C4	1.5 (4)
O2—S1—C8—Cl2	−63.88 (14)	C2—C3—C4—C5	−1.8 (5)
O2—S1—C8—C7	56.55 (17)	C3—C4—C5—C6	−0.3 (4)
O3—S1—C8—Cl1	−55.78 (15)	C4—C5—C6—C1	2.8 (4)
O3—S1—C8—Cl2	65.83 (16)	C4—C5—C6—C7	−177.1 (2)
O3—S1—C8—C7	−173.73 (16)	C1—C6—C7—O1	170.6 (3)
N1—S1—C8—Cl1	58.88 (14)	C1—C6—C7—C8	−10.4 (3)
N1—S1—C8—Cl2	−179.52 (12)	C5—C6—C7—O1	−9.6 (4)
N1—S1—C8—C7	−59.08 (17)	C5—C6—C7—C8	169.4 (2)
S1—N1—C1—C2	156.00 (19)	O1—C7—C8—Cl1	103.2 (2)
S1—N1—C1—C6	−24.5 (3)	O1—C7—C8—Cl2	−20.0 (3)
C9—N1—C1—C2	−18.8 (4)	O1—C7—C8—S1	−138.4 (2)
C9—N1—C1—C6	160.7 (2)	C6—C7—C8—Cl1	−75.9 (2)
S1—N1—C9—C10	118.6 (2)	C6—C7—C8—Cl2	160.91 (17)
C1—N1—C9—C10	−66.7 (3)	C6—C7—C8—S1	42.5 (2)

Symmetry codes: (i) −*x*, −*y*+1, −*z*; (ii) *x*, −*y*+1/2, *z*−1/2; (iii) −*x*, *y*+1/2, −*z*+1/2; (iv) −*x*, −*y*+1, −*z*+1; (v) −*x*+1, *y*+1/2, −*z*+1/2; (vi) *x*, −*y*+3/2, *z*−1/2; (vii) *x*, −*y*+1/2, *z*+1/2; (viii) −*x*, *y*−1/2, −*z*+1/2; (ix) *x*, −*y*+3/2, *z*+1/2; (x) −*x*+1, *y*−1/2, −*z*+1/2.

### Hydrogen-bond geometry (Å, °)

**Table:**

*D*—H···*A*	*D*—H	H···*A*	*D*···*A*	*D*—H···*A*
C2—H2···O3^vii^	0.9300	2.4800	3.359 (3)	157.00
C10—H10C···O1^x^	0.9600	2.6000	3.445 (4)	147.00

Symmetry codes: (vii) *x*, −*y*+1/2, *z*+1/2; (x) −*x*+1, *y*−1/2, −*z*+1/2.
